# Defining Digital Game-Based Learning for Science, Technology, Engineering, and Mathematics: A New Perspective on Design and Developmental Research

**DOI:** 10.2196/20537

**Published:** 2021-02-19

**Authors:** Shahrul Affendi Ishak, Rosseni Din, Umi Azmah Hasran

**Affiliations:** 1 Science, Technology, Engineering, and Mathematics Enculturation Research Centre Faculty of Education Universiti Kebangsaan Malaysia Bangi Malaysia; 2 Fuel Cell Institute Universiti Kebangsaan Malaysia Bangi Malaysia

**Keywords:** digital game-based learning, STEM digital game, game development model, game design, design and developmental research

## Abstract

In the modern age, digital games are widely used as informal media for Science, Technology, Engineering, and Mathematics (STEM) education and medical therapy for game-based learning. Digital games provide learners with a graphical system of interaction that enhances scientific concepts within an enjoyable environment. The vastly increasing number of digital games produced in the market affects the quality of STEM digital games while requiring multidisciplinary expertise. This paper proposes a framework for STEM digital game-based learning encompassing input-process-output stages. Several studies from the early 2000s onward were reviewed to discuss and present a new perspective on a framework for the design and development of digital games, particularly for STEM. This proposed framework consists of digital game development as input, experience as a process, and constructs as output. This simple and precise framework will generate a universal product for various types of learners. It can thus be used as a guideline for game designers, developers, and experts to develop STEM digital games and achieve better learning outcomes.

## Introduction

The use of digital games for education has been identified as one of the global pedagogical approaches required for 21st century learners [1]. The intention is to make STEM (science, technology, engineering, and mathematics) education more interactive and interesting and enhance understanding of STEM concepts [2,3]. Individuals in any age range can learn through digital games. Ultimately, it is more practical to use such games in kindergarten to grade 12 education (primary and secondary school) to increase students’ levels of interest in STEM. An interactive gaming system uses advanced graphics and programming tools. STEM content can be gamified easily in the current digital era. Game designers and developers often collaborate with teachers and experts to construct good instructional games for STEM learning as a universal product. This type of pedagogical approach is known as digital game-based learning [4] or STEM digital game-based learning [5,6].

Using digital games to enhance STEM education is important. Their purpose is to allow scientific concepts to be visualized [7-9]. The STEM curriculum consists of multiple facts and concepts learners should understand and practice in their daily lives. Some may not be easy to learn using conventional teaching pedagogy [10]. Teachers need supporting learning material to visualize each concept, for which digital games can be the best tools. Digital games allow learners to interact with game mechanics in a virtual world, achieving the set goals as a result of the desire to win triggered by these games. This makes learners feel enjoyment while simultaneously enabling them to gain a better understanding of STEM content. Their interaction and involvement in experiencing and understanding the game while resolving problems in the virtual world provide a meaningful gaming experience [2,8,9]. Thus, the use of digital games not only enhances learning, develops skills, and increases the memorization and understanding of STEM, it also helps to maintain interest and create a STEM mindset [11].

**Figure 1:**
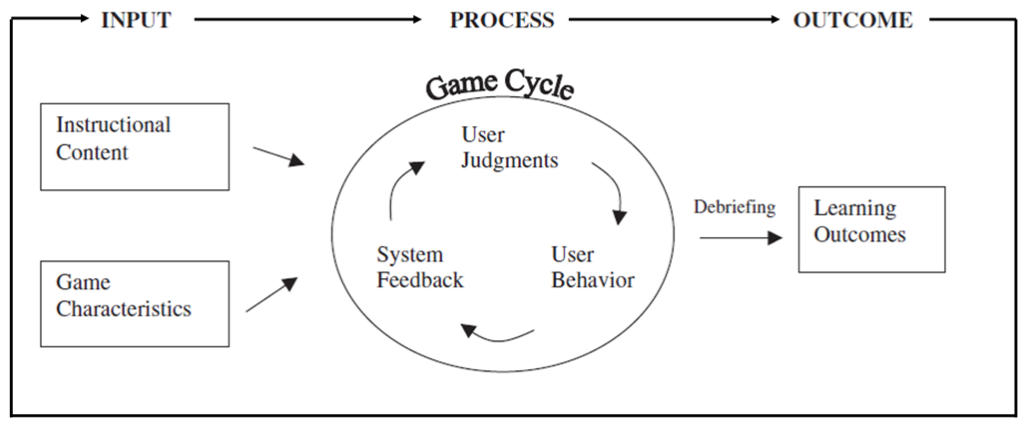
Input-process-output model adapted by Garris et al [13].

Digital games for formal and informal education are accessible through computers, tablets, and smartphones, all of which are popular among digital natives—the new digital generation [12]. STEM education is challenging for young people. Most digital games aim to enhance STEM learning on various gaming platforms [13]. This was introduced in the earliest model of game-based learning developed by Garris et al [13], which consisted of input, process, and output (Figure 1). Current studies on gaming and learning have 3 major and distinct perspectives—(1) research on input: how to develop educational digital games; (2) research on process: how the games work for users; and (3) research on outcome: impact of the games. Advancing gaming technology means the number of digital games keeps increasing, and developers are competing with each other [14]. Developing digital games for STEM involves a multitude of team experts. These collaborations lead to an agile form of development that requires multiple lightweight processes over a shorter period. This makes it easier for each game designer, game developer, and STEM expert to ensure the digital game undergoes a systematic development process suitable for STEM digital game-based learning.

**Figure 2:**
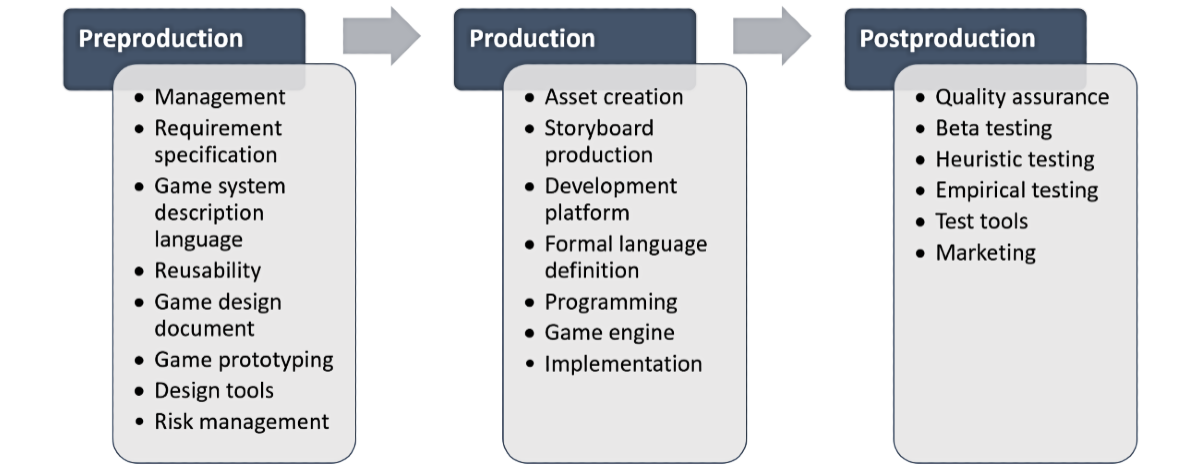
Game development process from industrial practice.

Game development processes are integrated and mapped into the design and development research, with emphasis on a systematic process of product development to achieve STEM learning outcomes. Digital game development undergoes iteration processes. From an industrial perspective, this involves preproduction, production, and postproduction phases as a linear process (Figure 2) [15]. However, studies [16-25] involving design and development research indicate that research products should undergo iteration processes until the development objective is achieved. Thus, digital games must be created for use by numerous people regardless of their abilities and demographic characteristics. This concept is synonymous with personalized learning based on a universal design for learning [16,17]. From an educational perspective, digital games are viewed as a learning tool in which STEM content is important in ensuring each game enhances learning and interest in STEM. Although previous studies [2,4,5,8,10,12,13,26-32] have introduced various models of digital game-based learning for specific outcomes, they have not described the development input-process-output structure as a holistic framework.

In the fourth industrial revolution environment, daily human activities have been influenced by digital technology. The new digital generation play digital games as part of their leisure activities. Numerous studies [26,27] on digital games have been conducted over the past two decades and have had a major influence on individual learning process; however, they have rarely discussed STEM learning. Because the number of digital games for STEM keeps increasing as a result of rapid development by the gaming industries, a relevant theoretical and practical underpinning of the development process for better STEM learning outcomes is required. As a universal product, the use of digital games for STEM does not rely on achieving better learning outcomes alone, it also needs to stimulate young people’s interest in STEM as a future career from an early age. Thus, this paper proposes a framework to understand STEM digital game-based learning encompassing design and development, gaming experience, and the generation of STEM outcomes.

## Digital Game-Based Learning for STEM

Discussions on the use of digital games for STEM can be confusing. It is a multidisciplinary field consisting of 2 different domains: STEM education and digital gaming. The link between these fields triggers the need for further research to understand how and why digital games affect STEM learning. Thus, the use of digital games and STEM learning should be studied in parallel. Digital game-based learning for STEM is also a difficult concept to define. In terms of their separate fields, digital game-based learning is defined as the use of digital games to enhance learning. Thus, to develop a more holistic perspective, we define STEM digital game-based learning as the process of creating an interactive STEM environment through digital games to enhance individual STEM learning. As such, several relevant design and development studies are now reviewed.

Most of the development models for any product undergo a systematic process. Several studies proposed a new model adapted from the original ADDIE (Analysis, Design, Development, Implementation, and Evaluation) model [20,33,34]. Din [18] revealed that the ADDIE model did not consist of a usability test, which is the most important process in product development. Other scholars have also adapted and improved the model to ensure a better systematic process [20,33,34]; however, in the context of developing a digital game, every process undergoes rapid and, some may argue, agile development. This is due to the higher number of digital games produced each day. Gaming industries tend to develop digital games in a shorter time, and some might not follow the systematic process proposed by the literature. This increases the number of low-quality digital games for users.

Din initially developed the Model *Pembangunan Sistem* to design and create a computer conferencing system [18] and further developed the model into Model *RekaBangun Sistem Pengajaran dan Pembelajaran* [19] after testing it in the development and validation of integrated meaningful hybrid e-training for computer science—a theoretical- and empirical-based design and development approach [20]. This model was tested in numerous other studies to generate the fourth version, the Model *RekaBangun Sistem Pengajaran dan Pembelajaran IV* now called the Model *RekaBangun SPP IV* [19]. Between 2011 and 2015, Din et al [16] rigorously tested the model and developed the Universal Design for Learning Instructional Design model. Between 2015 and 2020, Din combined this model with a simpler agile version of the Model *RekaBangun SPP IV* after rigorous testing on more than 15 online and mobile modules, systems, and apps [17]. This final and latest version comes complete with a universal design for learning and agile development method and contains modeling stages with value integration [17,21].

## Input as Digital Game Development

In digital game design, designers first identify the input elements. Universal design ensures a high degree of usability and accessible digital games for various types of learners regardless of ability [28,35,36]. Several models have been analyzed and proposed to determine which specific attributes are needed. A game perspective emphasizes content and game characteristics for enjoyment, while educational technology emphasizes learning content and a pedagogical approach. The combination of these 2 perspectives produces a better attribute as input for the game design system.

Gamification is well-known in educational technology as defining the process of integrating learning content into game mechanics [37]. The purpose is to make the learning content more interesting and engaging and to motivate learners throughout all elements of game-design [38]. Studies [6,13,17,19,22,28-30,39,40] indicate that several universal design attributes need to be incorporated into educational games such as learning theory, learning strategy, pedagogy, learning content, and game characteristics such as value integration. For STEM digital games, *STEM learning content* is a more suitable term representing the learning content attribute. All these attributes are subsequently included in the game design system.

**Figure 3:**
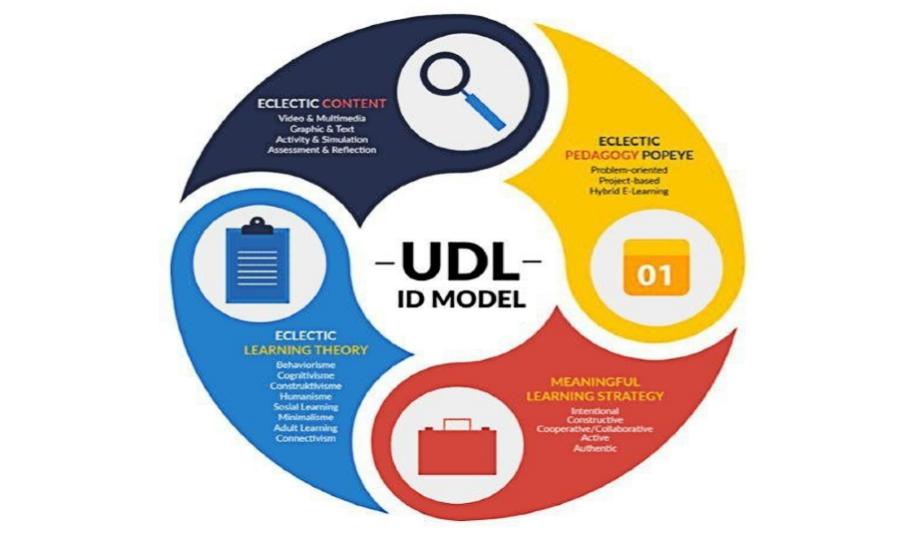
Eclectic Universal Design for Learning model.

**Figure 4:**
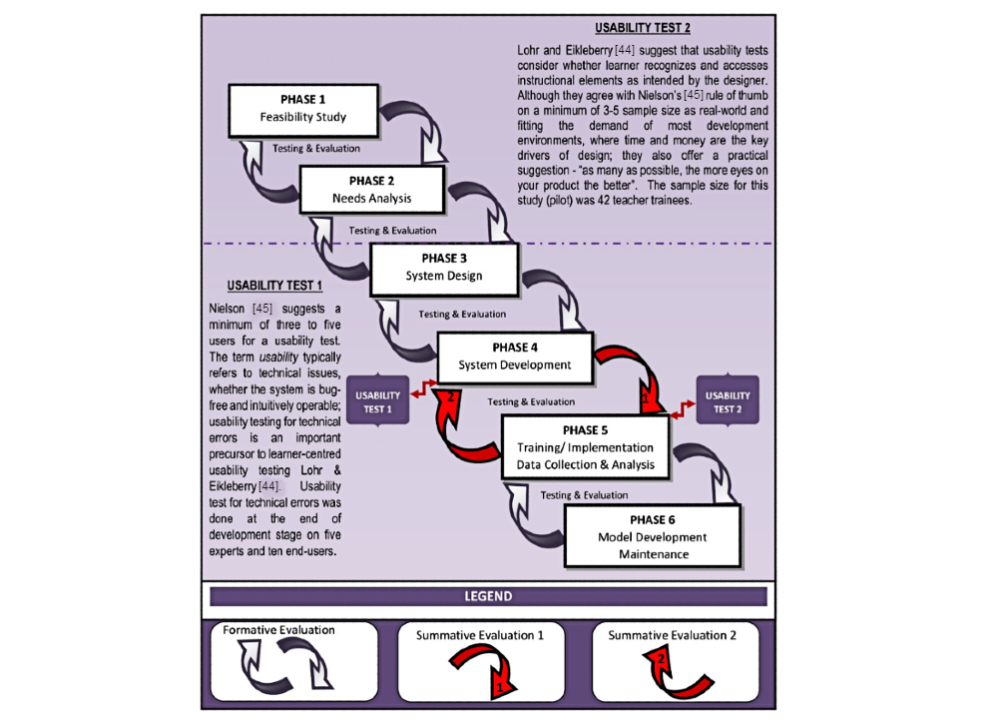
The development model [44,45].

Input for digital game development requires several attributes to undergo systematic development processes. These are integrated from digital games and education perspectives [17,19,30,39-43]. Din’s early development model [18-20] did not separate instructional design from the development model. Instead, instructional design processes were embedded into phases of development [19]. After 2015, Din extracted instructional design components from the development phases. This yielded 2 separate models, the instructional design model (Eclectic Universal Design for Learning model) presented in Figure 3 and the development model presented in Figure 4 [17,44]. The learning design is inspired by the universal design for learning model. It represents the expansion of phase 1 through phase 3 of the development model. The original model consisted of 4 main components: (1) eclectic learning theory, (2) eclectic content, (3) eclectic pedagogy, and (4) eclectic learning strategy.

**Figure 5:**
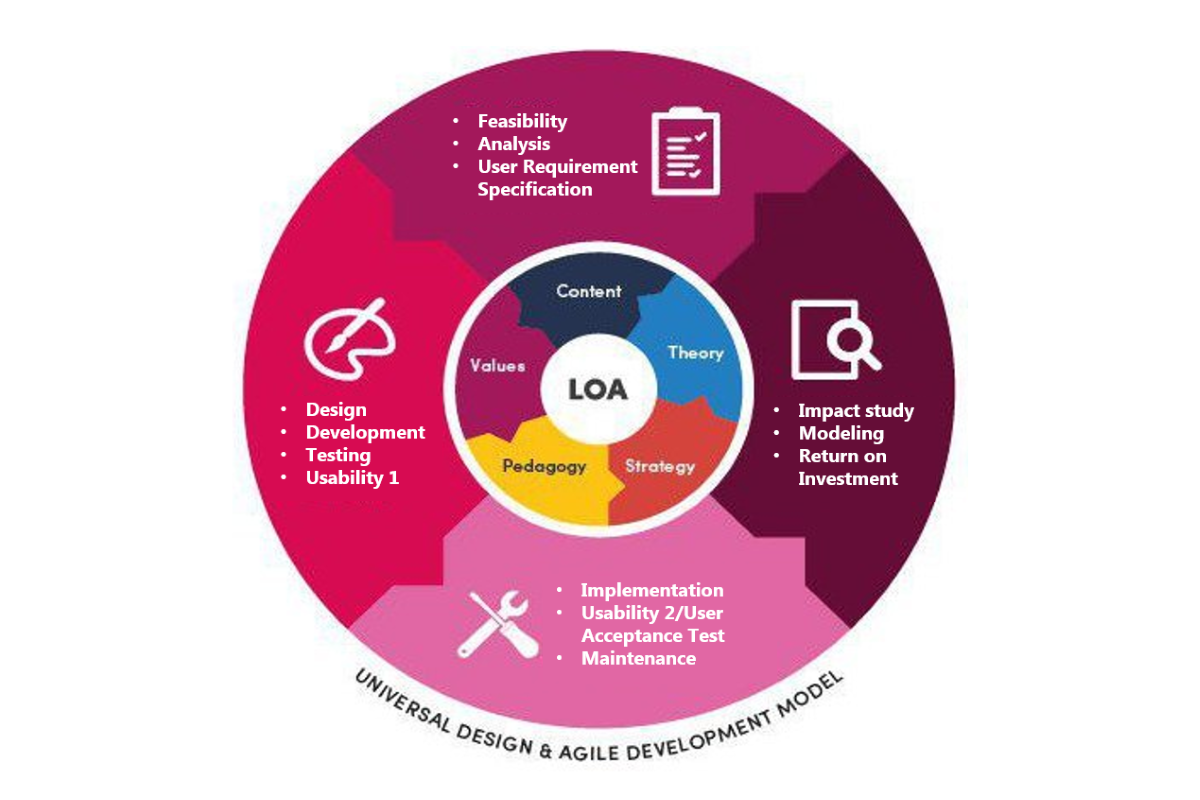
The final UDin model—2 decade transformation of the design, development, and modeling of a learning system.

The agile version [17,46] of the integrated model for the instructional design, development, and modeling of a personalized learning environment in education, namely the UDin model, is presented in Figure 5 [46]. According to Din, the UDin model, which is a 20-year transformation model of the design, development, and modeling of a learning system, added the taken-for-granted Learning Outcome component. This was aligned with the Assessment component and both were placed in the center as the innermost part of the model labeled Learning Outcome and Assessment [17,46]. The model emphasizes continuous assessment. The rubrics are mainly used as assessment tools. The assessment methods range from gamification, reflection, and visual-, video- and technology-mediated communication to field work [17,46]. Din’s previous work discusses the assessment method and tools in detail; this encompasses studies by Din et al [23-25,47], Azizul and Din [48], Batainah et al [49], Salleh et al [50], and Abdul Manaf et al [51]. Conventional quizzes transformed into interactive online quizzes are also used as formative evaluation alternatives in online modules. Some courses also retain the pen-and-pencil test, primarily for final summative evaluation.

**Figure 6:**
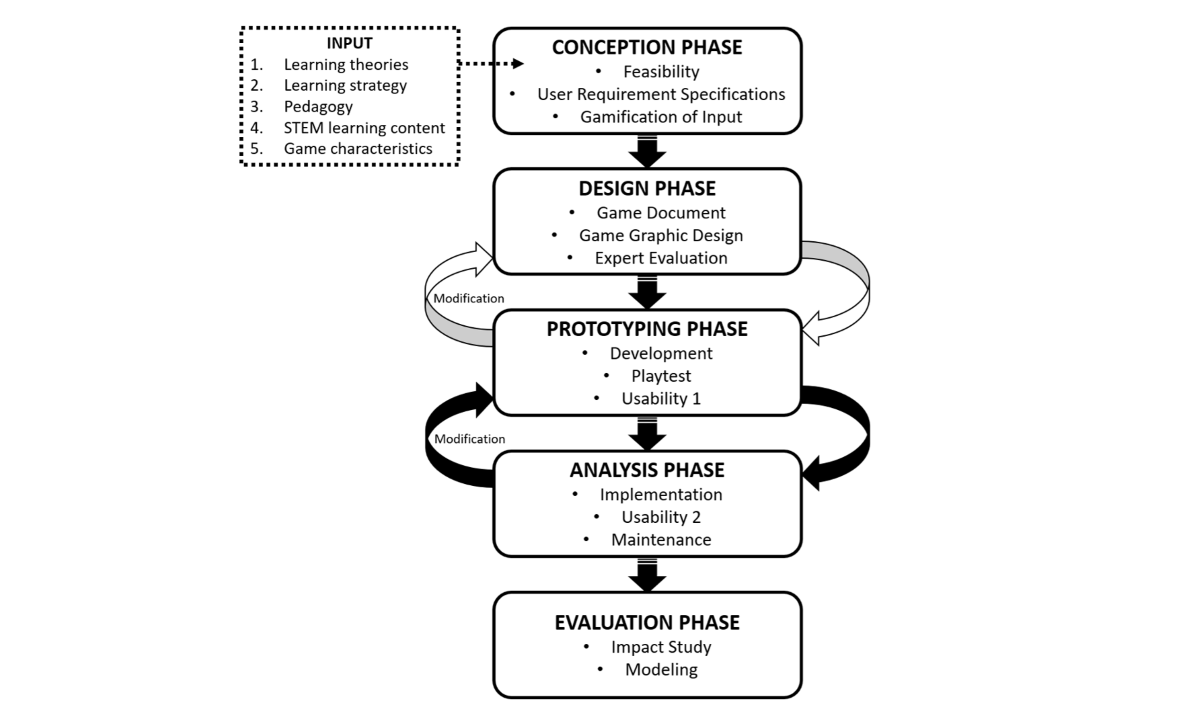
Proposed STEM digital game development process.

The simplified version of the STEM digital game development process is presented in Figure 6. As a product for research purposes, the framework consists of 5 main phases: conception phase, design phase, prototyping phase, analyzing phase, and evaluation phase. The conception phase starts with the feasibility of the proposed idea or game concept. Research needs to be undertaken by the developer or game designer to ensure the novelty of the idea compared with existing games. The game developer and STEM experts must therefore examine, analyze, and discuss the main concept of the game that needs to be developed. All 5 components of the universal design for learning mentioned in the input session adapting the UDin model [17,46] are followed in this conception phase. The adapted universal input attributes are learning theories, learning strategy, pedagogy, STEM learning content, and game characteristics. These are gamified into the mechanics of the game. Analysis of user requirement specifications ensures the game has a targeted audience. All discussion regarding the concept is contained in a game specification document that consists of team members, targeted users, game theme, game platform, game mechanics, and game concept.

The integration of input through gamification relies on theory. In the proposed model (Figure 6), learning theories serve to frame the context of STEM digital games. Several theories have been associated with digital game-based learning. However, to purposefully create STEM digital games for STEM education, 3 major learning theories have been identified: the theory of experiential learning, theory of self-determination, and the educational-psychology theory of interest development. Blending these theories with a learning strategy, pedagogy, STEM learning content, and game characteristics generates a holistic universal attribute to produce one digital game underlying learning and game development perspectives. The important element in creating STEM digital games is the integration of STEM content. This attribute plays a major role in characterizing STEM digital games. Next, the development of game graphic design is designed based on game specification document and later must to be evaluated by experts.

The prototyping phase is one in which the design and development of the digital games begins. The identified input attributes are ready for the graphics and programming process. All attributes act as an underlying basic or game design in a form of system and mechanics. To program the game system, game designers and programmers follow user-need specifications for the game design. The first prototype of the designed digital games undergoes a playtest among team members prior to a small-scale usability test with a targeted audience. This is to ensure that the digital game works without any major errors. Comments at this point are recorded for improvement. The prototyping phase continues with Usability Test 1 comprising a targeted audience of 3 to 5 users [45]. The games are then modified and improved based on the results.

The next phase is the analysis phase, in which the game is ready for large-scale testing. This consists of Usability Test 2, and all comments are recorded. However, if the result shows another emerging error, the game needs to undergo another prototyping phase so that improvements can be made. The development team need to ensure the iteration process between the prototyping and analysis phases meets the dateline and cost requirements. After the game is tested and data are collected, the programmer makes final improvements during the maintenance phase. The final phase is the evaluation phase in which the performance of the digital game is assessed. The evaluation measures the relevant constructs involved. It also determines whether the game has achieved the objective of development. Due to budget problems, the evaluation process sometimes starts simultaneously with Usability Test 2 [19,22]. In such cases, Usability Test 2 is conducted with a larger targeted audience so that evaluation can be performed at the end of the test.

## Process as Experience

User experience is one of the main components of using digital games in STEM education. During testing, there are interactions among users. Users enter the game cycle and make judgements based on observations to understand the system during the first attempt. The behavior of the game provokes the user to try what they have observed regarding the interactions taking place in the game system. The trial-and-error process allows formulation of a meta-framework of conceptual understanding as to how the system works in the developed digital game. The system feedback offered by the digital game due to the designed rules enables users to understand when certain actions do not produce the correct outcome. The cycle occurs continuously while users remain in the gaming world system.

The game cycle and STEM education are related. STEM education emphasizes the need for scientific thinking [11,52,53]. The digital game thus stimulates scientific thinking via observation (understanding how the game system works at the first attempt as a form of assumption), testing (making predictions and testing the assumption as a form of action), and drawing conclusions (system feedback shows whether the action performed produces the current movement inside the game). This game cycle provides the experience of engagement and involvement in an enjoyable environment. At the same time, the digital game helps users practice scientific skills without even realizing they are doing so.

## Output as Constructs Involved

**Figure 7:**
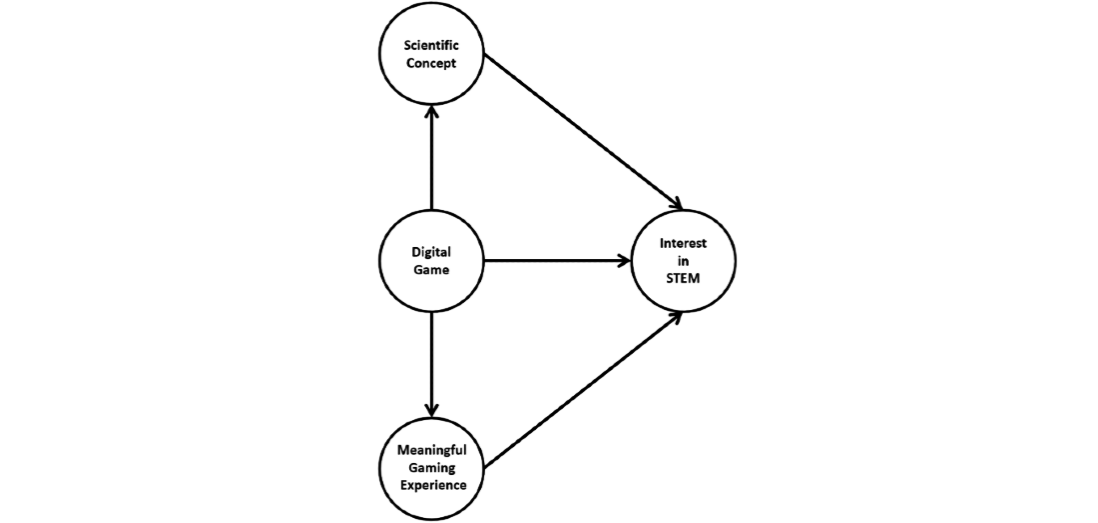
Proposed relationship of the constructs involve by using digital game.

There are several constructs involved when the user plays and interacts with the developed digital game. The constructs relate to the main objective of the digital game, which is designed according to STEM purposes. Research shows that any digital game provokes learning due to interaction via the game cycle, investigating the relationship between those constructs. Based on a review of literature, this paper identifies 4 major constructs:(1) digital game, (2) scientific concept, (3) meaningful gaming experience, and (4) interest in STEM (Figure 7). Figure 7 also indicates that the digital game influences interest in STEM through the presence of scientific concepts and meaningful gaming experience as mediators. The major constructs proposed in this paper need to be measured to produce the structural model and measure the effectiveness of STEM digital games.

To determine this relationship, several studies [2,9,11,31,32,54-79] were reviewed and the result mapped into one framework, as depicted in Figure 7. Because the data and results presented are scattered, this model purposely maps the result into the context of STEM digital games (Table 1). Based on the common findings, the digital game has a strong relationship with learning outcomes as the formation of scientific concepts is based on the game content. At the same time, research on digital games as entertainment also reveals a relationship with the gaming experience as the player feels a strong connection with the character inside the game world. However, Fisher [11] and other information sources [1,2,8,9,12,26,27,55-58,79] from the internet claim that the use of a digital game is the best pedagogical approach to increase or stimulate STEM interest among individuals. Digital games may therefore affect individual psychology in that the experience of playing is related to an interest in STEM. This relationship has been confirmed by Krapp [72] where individual interest is a process that undergoes 4 stages [72]. Providing STEM digital games is one of the practices based on Krapp’s concept of interest development. According to the literature, stimulating STEM interest using digital games is not a direct cause-effect relationship as another 2 variables have been identified as mediating this effect.

**Table 1:** Summary of research findings on the relationship between potential constructs as output for STEM digital games.

Variables	Findings	References
Digital games → scientific concepts	Digital games are potential tools for teaching STEM^a^ concepts via a specific topic. Knowledge gains underlie the gameplay. The integration of scientific content acts as a vehicle for gameplay. Most players exhibit higher knowledge gain, as a STEM learning outcome, using digital games. The target group, with the most potential for STEM digital games, is children.	[9,31,32,54-61]
Digital games → meaningful gaming experience	Playing digital games provides a gaming experience. As part of edutainment, good digital games create meaningful experiences for players. Players with a higher level of game experience and feedback engage in a more in-depth reflective process. Players will feel the fun element and have strong connections with the character played inside the game world.	[62-69]
Digital games → interest in STEM	Digital media have a significant relationship with an interest in STEM. Due to better gameplay, digital games might therefore be successful in increasing students’ interest in STEM. Studies show that using digital games may lead to a positive attitude toward STEM subjects and a STEM career.	[2,11,55,70-72]
Scientific concepts → interest in STEM	Knowledge gained by providing authentic scientific inquiry will trigger an interest in STEM. Students who play video games will engage in a STEM subject related to their lives.	[55,73-75]
Meaningful gaming experience → interest in STEM	Providing digital games will serve to enhance individual curiosity. Once attracted to the game, the playing process will increase the level of interest in the content gained. Because a meaningful gaming experience is a product of playing digital games, studies show that this factor is significantly related to an interest in STEM.	[76-79]

^a^STEM: science, technology, engineering, and mathematics.

To measure the relationship between the 4 identified variables (digital games, scientific concepts, meaningful gaming experience, and interest in STEM) simultaneously, these variables need to be defined and measured individually in the form of a measurement model. Structural equation modeling helps to measure and fit this hypothetical model with data in one go. This outcome at this stage is best achieved using smart-PLS software (SmartPLS GmbH) since it is at an exploratory stage.

**Figure 8:**
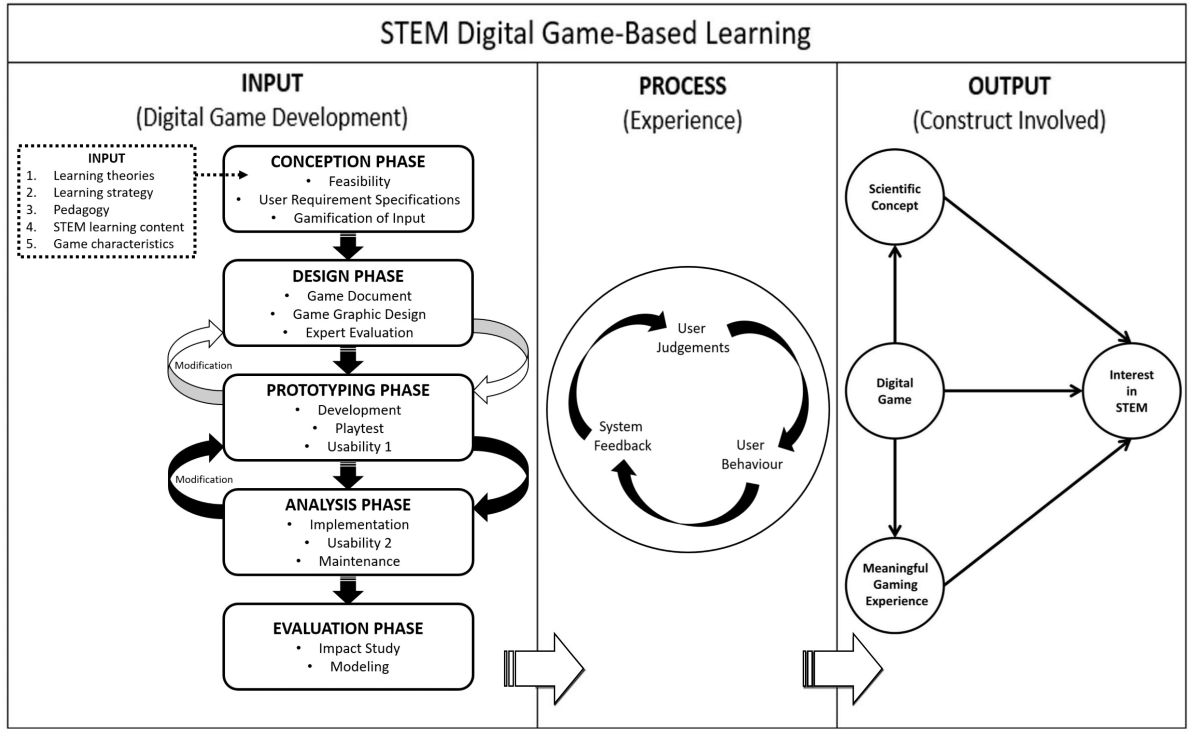
Proposed framework on STEM digital game-based learning.

The proposed STEM digital game-based learning framework is that of a linear input-process-output structure (Figure 8). This framework can be employed by any game designer, developer, or even teacher to develop a good STEM digital game with the objective of increasing STEM interest among learners. Young people with a low interest in STEM will therefore benefit from playing a STEM digital game.

## Conclusion

This paper proposes a framework for STEM digital game-based learning based on the current and vast body of gaming technology and a universal design for learning instructional design and agile development processes. Following the proposed model will enable the product to compete with what is available in a market where most developers are competing to produce digital games. However, the game needs to undergo a systematic development process as proposed. This paper thus outlined the specifications needed for a digital game to obtain the desired outcomes.

## Abbreviations

STEM: science, technology, engineering, and mathematics
